# A Complicated Case of Labor—Placenta Previa—Hydramnios Fatty Degeneration of the Placenta

**Published:** 1894-08

**Authors:** V. D. Lockhart

**Affiliations:** Homer, Ga.


### A COMPLICATED CASE OF LABOR—PLACENTA PREVIA—HYDRAMNIOS FATTY DEGENERATION OF THE PLACENTA.

BY V. D. LOCKHART, M.D., HOMER, GA.

That nature should reverse itself, so that "the first shall be last and the last shall be first," does not appear to be common in obstetrical work, yet it so happened to me recently in a case of labor in which the placenta was actually expelled in mass before the fetal head had taken the first step in the wonderful process of delivery. It was a case, too, which confirms the saying of one of our modern authors that where one thing goes wrong in a case of labor, we may expect new complications to reveal themselves at almost every stage.

Mrs. D——, thirty-four years old, mother of two healthy children; family history free from any specific disease.

June 22 last, she consulted me at my office, stating that she was six months in pregnancy. I had attended her in confinement three years ago, and she had had one abortion since, but no physician had been called at that time.

She complained of unusual sensations in her abdomen, swelling of the feet and some dizziness. The urine was free from albumen, and she was not at all anemic. The action of the heart was at times quite irregular, but no organic disease was discovered. From her appearance and size of the abdomen, I suggested that she might be mistaken, and that she might be then at least eight months in pregnancy, but to this she demurred, and said she knew she was swollen by some dropsical effusion. In reply to my questions on the point, she said there was an enormous amount of fluid expelled at the time of the last abortion, and as I was sure that the fluid was not in the abdominal cavity, I suspected hydramnios. She had also had occasional hemorrhages, and recently one of these hemorrhages had been alarming on account of its sudden onset, but so far there had been no expulsive pain. I communicated my fears to her husband, and advised the patient to send for me as soon as another hemorrhage occurred, as I was almost certain I had a case of placenta previa on my hands.

Her general symptoms improved some, and all went well until July 17, nearly one month subsequent to her visit to my office, when, in packing away some bed covers, she was again taken with hemorrhage about one o'clock P. M., followed by expulsive labor-pains soon thereafter. The flow of blood was quite rapid, and she soon became very weak. The family lived seven miles from town, and I was out visiting other patients when the messenger came, so that it was quite dark before I reached the house.

Upon examination, I found the vagina filled with an enormously distended bag of water, which had arrested the hemorrhage in a great measure by mechanical pressure, so that instead of a gush of blood at each pain, as we usually have in such cases, there was a smart oozing between the pains. I could feel the free edge of the placenta on the left side of the uterus. I could not feel the fetus above, or tell anything about the presentation. I decided to wait awhile and watch the progress of the case, and in about forty minutes the sac of amniotic fluid had collected in such quantity as to effectually obstruct the labor; so taking a lead pencil in my left hand between the thumb and forefinger, I held it to the presenting part, and at the next pain the membranes ruptured, and such a flow of water I have never seen. It came with quite a loud, rushing sound and flowed down nearly all over the bed and bedding, so as to run off at the sides, wetting the woman thoroughly. I think there must have been as much as a pailful—two gallons or more. Hasty examination was now made with a view to completing the labor by a turning operation if necessary, but I now found the placenta lying loosely in front of the fetus. I could move it from side to side with my finger. The uterus had not contracted, so I made firm pressure over the fundus, and in a few minutes the placenta was expelled in mass, hanging by the membranes, together with the cord.

I could now feel the fetal head in the r. o. a. position, and advancing with each pain, so I rejoiced to know that my troubles were so soon to end. The labor proceeded naturally from that stage, and in twenty minutes the fetus was lying on the bed with the placenta and entirely enveloped within the membranes, which I quickly opened to find a well developed seven months' child apparently dead for several days.

Upon further examination, I found the cord and membranes about normal in appearance, but the maternal surface of the placenta had undergone fatty degeneration; in fact, it was apparently a mass of fat, except at one edge, a spot the size of the palm of the hand, where all the hemorrhages had taken place, and which had been the last to become detached. The child had evidently perished from lack of blood supply, and it was to me a wonder how it had survived so long, and continued to grow through so small an area of placental attachment.

The placenta was about normal in size, and had been well nourished at one time, but its structure had been undermined by degrees until it had dropped from the stem, so to speak.

I have seen cases of placenta previa, and cases of hydramnios, too, and I have sometimes seen cases of degeneration of the placenta—in fact, it is frequently the cause of abortion—but I have never seen such a complication of troubles, or read of them in any of the books, and I therefore send it to your JOURNAL in order that it may go on record as one of the curiosities of obstetrics.

